# Supplementary material for: DrugComb update: a more comprehensive drug sensitivity data repository and analysis portal
Source: Nucleic Acids Res. 2021 Jun 1;49(W1):W174–84. doi: 10.1093/nar/gkab438 (PMC8218202; doi:10.1093/nar/gkab438)
Supplement: gkab438_Supplemental_File [file gkab438_supplemental_file.pdf]

Supplementary Table 1. Database sources of DrugComb. Study names are determined by first authors of the publications or by the names of databases where the datasets were first deposited. Newly curated datasets are shown in the shaded areas as compared to the first four datasets that were deposited in the original version.

Study name	Disease	Data source	Pubmed ID	Number of drugs	Number of blocks	Number of cell lines	Number of tissues	Full dose-response matrix size
ONEIL	Cancer	Publication	26983881	38	92208	39	6	5x5
CLOUD	Cancer	Publication	28530711	283	40160	1	1	2x2
ALMANAC	Cancer	Publication	28446463	103	311604	60	9	4x4, 4x6
FORCINA	Cancer	Publication	28601558	1818	1818	1	1	2x2
NCATS_ATL	Cancer	NCATS Tripod		22	30	1	1	10x10
MATHEWS	Cancer	NCATS Tripod	24469833	477	1119	1	1	6x6, 10x10
NCATS_DIPG	Cancer	NCATS Tripod		2450	8854	2	2	6x6, 10x10
NCATS_ES(FAKI/AURKI)	Cancer	NCATS Tripod		1909	1910	1	1	6x6
NCATS_ES(NAMPT+PARP)	Cancer	NCATS Tripod		94	4628	4	3	6x6, 10x10
WILSON	Cancer	NCATS Tripod	30289729	31	764	2	1	6x6, 10x10
NCATS_HL	Cancer	NCATS Tripod		1910	2694	4	2	6x6, 10x10
YOHE	Cancer	NCATS Tripod	29973406	25	270	3	2	10x10
NCATS_2D_3D	Cancer	NCATS Tripod		5	70	2	2	10x10
PHELAN	Cancer	NCATS Tripod	29925955	16	62	1	1	10x10
NCATS_MDR_CS	Cancer	NCATS Tripod		18	68	2	1	10x10
CCLE	Cancer	PharmacoDB	22460905	24	11670	503	24	6x1, 7x1, 8x1
CTRPV2	Cancer	PharmacoDB	26482930	544	395263	887	24	8x1 ~ 29x1
FIMM	Cancer	PharmacoDB	24056683	52	2561	50	5	5x1
GCSI	Cancer	PharmacoDB	27193678	16	6455	409	23	8x1, 9x1
GDSC1	Cancer	PharmacoDB	23180760	250	225480	1074	30	5x1, 9x1
GRAY	Cancer	PharmacoDB	24176112	89	9413	70	2	9x1
UHNBREAST	Cancer	PharmacoDB	26771497	4	52	15	1	9x1, 18x1
BEATAML	Cancer	Publication	30333627	122	59348	528	1	7x1
FLOBAK	Cancer	Publication	31664030	19	9984	8	7	6x6
ASTRAZENECA	Cancer	AstraZeneca	31209238	116	20482	153	10	6x6
FRIEDMAN	Cancer	Publication	26461489	108	208008	36	1	3x3
SCHMIDT	Cancer	Publication	24101737	4	100	5	1	8x8
MILLER	Cancer	Publication	24065146	13	82	1	1	8x8
FRIEDMAN2	Cancer	Publication	28446504	76	28500	10	1	3x3
TOURET	SARS-CoV-2	Publication	32753646	1516	1520	1	1	1x1
GORDON	SARS-CoV-2	Publication	32353859	75	290	1	1	5x1, 6x1, 7x1
ELLINGER	SARS-CoV-2	ChEMBL		5604	5632	1	1	1x1
MOTT	Malaria	NCATS Tripod	26403635	223	17072	3	1	6x6, 10x10
NCATS_SARS-COV-2DPI	SARS-CoV-2	NCATS Tripod		56	206	1	1	6x6
BOBROWSKI	SARS-CoV-2	NCATS Tripod	32637956	34	262	1	1	6x6
DYALL	Ebola	NCATS Tripod	29939303	17	432	2	2	6x6
FALLAHI-SICHANI	Cancer	Publication	28069687	10	111	5	1	10x1, 20x1

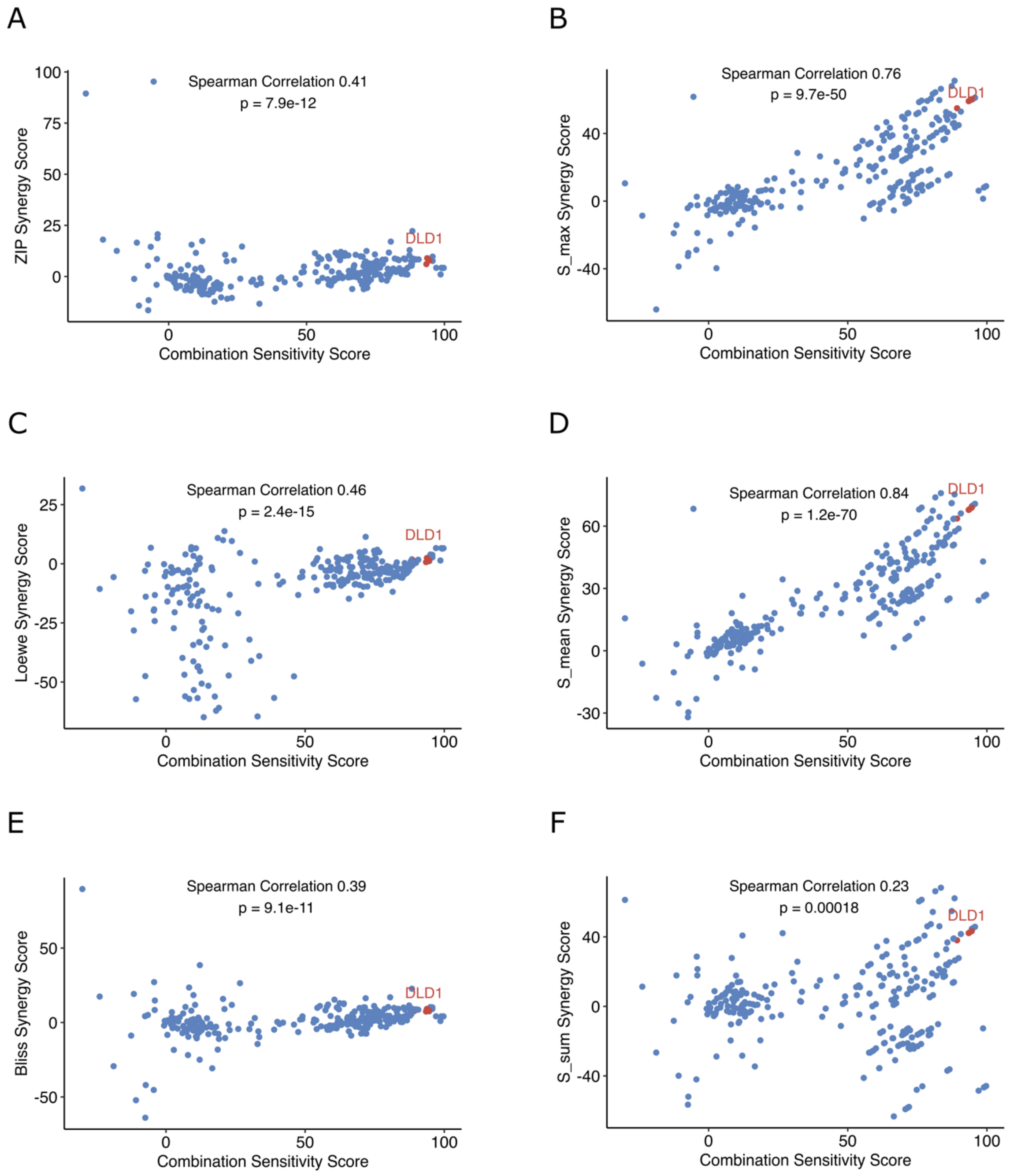

Supplementary Figure 1. SS plots for vorinostat and sorafenib combination across 128 cell lines. ZIP, Leowe, Bliss, as well as three S scores are plotted with CSS score. DLD-1 is a colon cancer cell line, which has shown strong synergy and sensitivity to the combination.
